# Adipose Tissue-Derived Stem Cell Extracellular Vesicles Suppress Glioblastoma Proliferation, Invasiveness and Angiogenesis

**DOI:** 10.3390/cells12091247

**Published:** 2023-04-25

**Authors:** Dovydas Gečys, Rūta Skredėnienė, Emilija Gečytė, Arūnas Kazlauskas, Ingrida Balnytė, Aistė Jekabsone

**Affiliations:** 1 Institute of Pharmaceutical Technologies, Faculty of Pharmacy, Lithuanian University of Health Sciences, LT-50162 Kaunas, Lithuania; 2 Laboratory of Molecular Cardiology, Institute of Cardiology, Lithuanian University of Health Sciences, LT-50162 Kaunas, Lithuania; 3 Department of Histology and Embryology, Faculty of Medicine, Lithuanian University of Health Sciences, LT-44307 Kaunas, Lithuania; 4 Laboratory of Molecular Neurooncology, Neuroscience Institute, Lithuanian University of Health Sciences, LT-50161 Kaunas, Lithuania; 5 Preclinical Research Laboratory for Medicinal Products, Institute of Cardiology, Lithuanian University of Health Sciences, LT-50162 Kaunas, Lithuania

**Keywords:** adipose-derived mesenchymal stem cells, extracellular vesicles, gene expression, chorioallantoic membrane model

## Abstract

Extracellular vesicles (EVs) are attractive anticancer drug delivery candidates as they confer several fundamental properties, such as low immunogenicity and the ability to cross biological barriers. Mesenchymal stem cells (MSCs) are convenient producers for high EV yields, and patient-derived adipose tissue MSC-EVs could serve as personalised carriers. However, MSC-EV applications raise critical concerns as their natural cargo can affect tumour progression in both inducing and suppressing ways. In this study, we investigated the effect of adipose tissue-derived mesenchymal stem cell EVs (ASC-EVs) on several glioblastoma (GBM) cell lines to define their applicability for anticancer therapies. ASC-EVs were isolated from a cell-conditioned medium and characterised by size and specific markers. The internalisation of fluorescently labelled ASC-EVs by human GBM cells HROG36, U87 MG, and T98G was evaluated by fluorescent microscopy. Changes in GBM cell proliferation after ASC-EV application were determined by the metabolic PrestoBlue assay. Expression alterations in genes responsible for cell adhesion, proliferation, migration, and angiogenesis were evaluated by quantitative real-time PCR. ASC-EV effects on tumour invasiveness and neoangiogenesis in ovo were analysed on the chicken embryo chorioallantoic membrane model (CAM). ASC-EV treatment reduced GBM proliferation in vitro and significantly downregulated invasiveness-related genes ITGα5 (in T98G and HROG63) and ITGβ3 (in HROG36) and the vascularisation-inducing gene KDR (in all GBM lines). Additionally, an approximate 65% reduction in the GBM invasion rate was observed in CAM after ASC-EV treatment. Our study indicates that ASC-EVs possess antitumour properties, reducing GBM cell proliferation and invasiveness, and can be applied as anticancer therapeutics and medicine carriers.

## 1. Introduction

Cancer remains one of the leading causes of mortality worldwide [1]. Glioblastoma (GBM) is classified as a grade IV central nervous system tumour by the World Health Organization (WHO) and is usually characterised by a poor prognosis and low survival rate [2]. Although several treatment options are available, including surgery, chemotherapy, radiotherapy, immunotherapy, and targeted therapy, patients generally survive up to 14 months after diagnosis [3]. CRIPSR-Cas9-modified CAR T cell therapies revolutionising leukaemia treatment appeared not so efficient on solid tumours, including GBM [4]. Some preclinical tests show promising results [5,6]; however, clinical trial results are not encouraging [7]. The treatment of GBM remains a complex challenge, as previous efforts to establish improved therapies resulted in a very modest survival increase, keeping the 5-year GBM survival rate below 10% [8].

MSCs are stromal cells that hold the capacity to self-renew as well as display multi-lineage differentiation. MSCs can be harvested from various tissues, including the umbilical cord, menses blood, bone marrow, and adipose tissue [9]. Notably, MSCs have affinity and natural tropism towards tumour sites and can modulate multiple biological processes related to cancer, such as angiogenesis, migration, and epithelial-mesenchymal transition [10]. Additionally, recent studies show that membranous extracellular vesicles (EVs) secreted by MSCs could be vital in developing novel therapeutic strategies for GBM. In particular, exosomes, a subtype of EVs, sizing 40–160 nm in diameter, can affect physiological homeostasis or the progression of human diseases, including cancer [11,12]. Exosomes are natural mediators of intercellular communication and comprise complex cargoes such as lipids, proteins, and nucleic acids, including DNA, mRNA, and non-coding RNAs [13,14]. It is important to emphasise that MSC-derived exosomes can function as essential regulators of the tumour microenvironment and modulate oncogenic processes such as proliferation, migration, and angiogenesis [11]. Additionally, studies show that MSC-derived exosomes are involved in cancer therapy resistance [12]. EVs, as a novel therapeutic strategy, possess several attractive properties, including the ability to cross the blood–brain barrier [15], lack of cytotoxicity for healthy cells [16], exceptional biocompatibility, as well as the potential for modification and the ability to be loaded with exogenous compounds such as chemotherapy drugs [17,18]. 

However, MSC-derived EV application in cancer therapy raises critical concerns as they are primarily studied for regenerative medicine approaches and possess proliferation-stimulating capacity, which might stimulate tumour growth [19]. Experimental evidence is controversial and shows that MSC-derived EVs can affect tumour progression in both promoting and suppressing ways. It was reported that exosomes from MSCs induce antitumour effects in prostate, bladder, renal, and liver cancer in vitro models [20,21]. However, several studies found that the same exosomes could promote breast cancer [22,23], gastric cancer [24,25], and bone cancer [24] cell proliferation and migration. Despite rapidly gaining popularity among researchers, the effect of natural MSC-derived EVs on glioma cells have not been widely studied. MSC-derived exosomes inhibited tumour progression when loaded with miR-133b or miR-146b [26,27]; however, without the additional cargo, they increased the proliferation and self-renewal of glioma stem cells [28]. On the other hand, MSCs are shown to inhibit angiogenesis [29] and induce glioma cell senescence and differentiation in a paracrine manner [30].

In this study, we aimed to evaluate the effects of adipose tissue-derived mesenchymal stem cell exosomes (ASC-EVs) on the proliferation and expression of genes related to tumour progression and migration in GBM cell cultures, as well as examine ASC-EV impact on angiogenesis and GBM invasiveness in ovo. 

## 2. Materials and Methods

### 2.1. Cell Cultures

For EV collection, ASC/hTERT1 (Evercyte, Vienna, Austria) cells were cultivated using Endothelial Cell Growth Medium-2 medium (Lonza, Basel, Switzerland) supplemented with 2% FBS (Gibco, Thermo Fisher Scientific, Bleiswijk, The Netherlands), and hydrocortisone, hFGF-B, VEGF, R3-IGF, Ascorbic Acid, hEGF, Heparin, GA-1000 (Lonza, Basel, Switzerland). For in vivo and in ovo experiments, GBM cell lines were cultivated in DMEM/F12 + Glutamax + 10% FBS (Gibco, Thermo Fisher Scientific, Bleiswijk, The Netherlands) for HROG36 (Cell Lines Service GmbH, Eppelheim, Germany); DMEM + Glutamax + 10% FBS (Gibco, Thermo Fisher Scientific, Bleiswijk, The Netherlands) for U87 MG (European Collection of Cell Cultures (ECACC, Salisbury, UK); aMEM + Glutamax + 10% FBS (Gibco, Thermo Fisher Scientific, Bleiswijk, The Netherlands) for T98G (ATCC, Manassas, VA, USA) cells. All mediums were supplemented with penicillin–streptomycin solution (Gibco, Thermo Fisher Scientific, Bleiswijk, The Netherlands). All cells were cultured in a 37 °C, 5% CO_2_ humidified incubator.

### 2.2. Extracellular Vesicle Isolation and Characterization

An ASC/hTERT1-conditioned medium was used to isolate EVs. When the cell growth reached 80–90% confluence, the growth medium was changed to a formulation supplemented with EV-depleted FBS (Gibco, Thermo Fisher Scientific, Bleiswijk, The Netherlands). After 48 h, the media were collected and filtered through 0.22 µm PVDF filters to eliminate larger particles. The solution containing EVs was mixed with Total Exosome Isolation Reagent (Invitrogen, Thermo Fisher Scientific, Bleiswijk, The Netherlands), and EVs were collected according to the manufacturer’s instruction. The pellet was reconstituted in 100 μL of PBS. Then, EV suspension was passed through the pre-design size exclusion chromatography (SEC) column (Exo-Spin, Cell Guidance Systems Ltd., Cambridge, UK) according to the manufacturer’s instruction to remove co-precipitated proteins. The Bradford assay (Sigma-Aldrich, Taufkirchen, Germany) was used to determine the quantity of total protein present in the particle isolate samples. Measurements were taken with Tecan Infinite 200 PRO plate reader (Tecan Austria GmbH, Grödig, Austria). Concentrations of CD9, CD63, CD81, cytochrome c (Cyt c), syntenin-1, and integrin α4β1 (also known as Very Late Antigen-4, VLA-4) in particle samples were determined using 6-Plex Human ProcartaPlex Panel (Thermo Fisher Scientific, Bleiswijk, The Netherlands) on Luminex 200 device (Luminex, Austin, TX, USA). The size distribution of particles present in the samples was analysed using two techniques. Nanoparticle tracking analysis (NTA) was performed using a Nanosight DS300 analyser (Malvern PANalytical, Malvern, UK) and dynamic light scatter (DLS) measurements were taken using a ZetaSizer analyser (ZetaSizer Nano ZS, Malvern PANalytical, UK).

### 2.3. Extracellular Vesicle Uptake Assay

HROG36, U87 MG, and T98G cells were seeded in 35 mm confocal Petri dishes and incubated for 24 h in the aforementioned growth medium for cells to attach. SYTO RNA Select Green fluorescent cell dye (Invitrogen, Thermo Fisher Scientific, Bleiswijk, The Netherlands) was used to label EVs, following the manufacturer’s protocol. The labelled particle samples were then purified to remove any unincorporated dye using SEC columns (Exo-Spin, Cell Guidance Systems Ltd., Cambridge, UK). Per Petri dish, 10 µg of total EV protein was used. The fluorescence of the stained particles before and after SEC was measured using a Qubit 3.0 fluorometer (Life Technologies, Thermo Fisher Scientific, Bleiswijk, The Netherlands). The particle samples were then transferred onto cells, and cell imaging was conducted using a Zeiss Axio Observer Z1 fluorescence imaging system (Zeiss, White Plains, NY, USA). The fluorescence intensity of the cells was calculated using ImageJ software (National Institutes of Health, Bethesda, MD, USA) [31].

### 2.4. Cell Proliferation Assessment

For evaluation of changes in cell proliferation, PrestoBlue Cell Viability Reagent (Thermo Fisher Scientific, Invitrogen, Bleiswijk, The Netherlands) was used. HROG36, U87 MG, and T98G cells (5000 cells per well) were seeded in 96-multiwell plates and treated with ASC-EVs. Following 24 h post-EV application, the growth medium was replaced to eliminate any uninternalised EVs, and the cells were incubated for another 48 h. Following a total incubation period of 72 h, a reaction mix consisting of 90 μL of growth medium supplemented with 10 μL of PrestoBlue reagent was added to the cells and incubated for 1 h. The fluorescence intensity of the plate wells was measured using a Tecan Infinite 200 PRO plate reader. The cell proliferation was represented as a percentage of the values observed in untreated cells.

### 2.5. GBM Model on Chicken Embryo Chorioallantoic Membrane

According to the law in force in the EU and Lithuania, no approval for studies using the Chicken Embryo Chorioallantoic Membrane (CAM) model is needed from the Ethics Committee. Fertilised chicken eggs (Cobb 500) were acquired from a local hatchery (Rumšiškės, Lithuania) and kept in an incubator (Maino incubators, Oltrona di San Mamette, Italy) at 37 °C temperature and 60% relative air humidity. For promoting embryo development, an automatic rotator was employed to roll the eggs once per hour until day 3 of embryo development (EDD3). On EDD3, the mechanical rotation was ceased, and the eggshells were cleansed with 70% ethanol. A small hole was drilled at the location of an air chamber, and roughly 2 mL of egg white was drawn using a sterile syringe to detach the CAM from the shell. Following this, a small square of approximately 1 cm^2^ was drilled, and the eggshell was cautiously removed. The created window was sealed with sterile parafilm. GBM cells were cultivated under aforementioned standard conditions with 5 μg/mL ASC-EVs in the growth medium for 48 h. After incubation, cells were trypsinised and resuspended in 20 µL of type I rat tail collagen (Gibco, Gaithersburg, MD, USA) per 1 × 10^6^ cells. Using a blade, pieces of 9 mm^3^ (3 × 3 × 1 mm) absorbable surgical sponge were formed (Surgispon, Aegis Lifesciences, New Delhi, India) and each piece was mixed with 20 µL of cell suspension. The sponges were implanted on the CAM near the main blood vessels on EDD7. The changes in tumour growth were monitored in vivo during EDD9-12, using a stereomicroscope (SZX2-RFA16, Olympus, Tokyo, Japan). The tumour images were captured by a digital camera (DP92, Olympus, Tokyo, Japan) and CellSens Dimension 1.9 digital imaging software. Following 5 days of incubation at EDD12, the samples were collected, fixed in a buffered 10% formalin solution for 24 h, and embedded in paraffin wax. Sections of 3 µm thickness were cut from the specimens using a microtome (Leica Microsystems Inc., Buffalo Grove, IL, USA) and stained with hematoxylin and eosin (H–E).

The H-E-stained CAM slides were visualised and photographed using a light microscope (BX40F4, Olympus, Tokyo, Japan) and a digital camera (XC30, Olympus, Tokyo, Japan) equipped with CellSens Dimension 1.9 software. The CAMs with tumours were divided into two categories: invasive and non-invasive. The invasion was defined as the destruction of the chorionic epithelium (ChE) and/or migration of tumour cells into the CAM mesenchyme while non-invasive tumours were present on the surface of the CAM without disrupting the integrity of the ChE. CAM thickness and the number of blood vessels were assessed by capturing images of the H-E-stained CAM directly under the tumour at 4× magnification. The CAM thickness (µm) was measured in ten areas, and the mean thickness was calculated in the area under the tumour. Only blood vessels with a diameter larger than 10 µm were counted.

### 2.6. Gene Expression Analysis

A commercial PureLink RNA extraction mini kit (Invitrogen, Thermo Fisher Scientific, Bleiswijk, The Netherlands) was used to extract total RNA from the cells. After RNA extraction, RNA samples were treated with DNAse I (Thermo Fisher Scientific, Vilnius, Lithuania) as per the manufacturer’s instructions and reverse transcribed with a High-Capacity Reverse Transcription kit (Life Technologies, Thermo Fisher Scientific, Bleiswijk, The Netherlands). Real-time PCR was performed using Power SYBR Green PCR mix under standard conditions on a 7900HT PCR system (Applied Biosystems, Foster City, CA, USA). PCR primers used are listed in Table A1. For gene expression data normalisation, β-actin and Glyceraldehyde 3-phosphate dehydrogenase (GAPDH) genes were used as endogenous controls. Alterations in gene expression were analysed using the 2^−∆∆ct^ method [32].

### 2.7. Statistical Analysis

Statistical analyses and visualisations were performed using GraphPad Prism 9 software (GraphPad Software Inc., San Diego, CA, USA). Data distribution was evaluated using the Shapiro–Wilk normality test. Quantitative differences between the two groups were evaluated by the Mann–Whitney U test.

## 3. Results

### 3.1. Identification and Characterisation of ASC-Derived EVs

**Figure 1:**
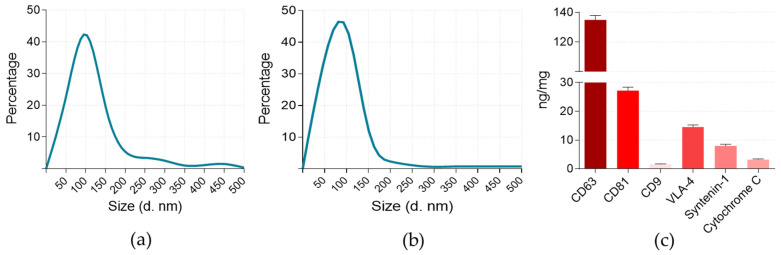
Characteristics of ASC-EVs. (**a**) NTA results. The graphs show particle sizes ranging from 50 to 450 nm, with a peak of 100 nm. (**b**) DLS results. Size measurements coincided with NTA data. (**c**) Multiplex EV marker assay confirmed the presence of tetraspanins CD63 and CD81, as well as adapter protein syntenin-1 and cell surface protein VLA-4. Results are given as a ratio of the target protein amount per mg of total EV protein.

At first, EVs collected from ASC cell-conditioned medium were evaluated for size distribution using NTA (Figure 1a). The samples contained particles ranging from 10 to 500 nm in diameter, with the highest peak at 100 nm. Particles in the 50–150 nm range accounted for 87% of the sample; the determined particle concentration in isolates was 4 × 10^10^ ± 1.2 × 10^9^/mL. NTA detected some particles greater than 220 nm in diameter which may be a result of particle aggregation. Despite this, particles < 220 nm in diameter constituted for 90% of the total sample. Next, particle samples were tested for the markers recommended for EV sample characterisation [33], including CD9, CD81, CD63, syntenin-1, and VLA-4 presence in the samples (Figure 1c). CD63 and CD81 are non-tissue-specific tetraspanins indicating the endosomal origin of EVs, together with the tissue-specific tetraspanin CD9. Syntenin-1 is an adapter protein involved in the trafficking of transmembrane proteins and exosome biogenesis [34]. VLA-4 is an adhesion molecule, an outer membrane cell surface marker [35]. The mitochondrial transmembrane space protein Cyt c represents the presence of EVs from other intracellular compartments better than a plasma membrane or endosomes. The analysis confirmed that all tested markers were present in the samples, with CD63 being the most abundant. However, only small traces of CD9 were found in the particle samples. This could be expected, as tetraspanin CD9 is observed to be at a considerably lower concentration in ASC-EVs and some other MSCs when compared to CD63 or CD81 [33,36].

### 3.2. Tracking of ASC-Derived EVs in GBM Cultures

**Figure 2:**
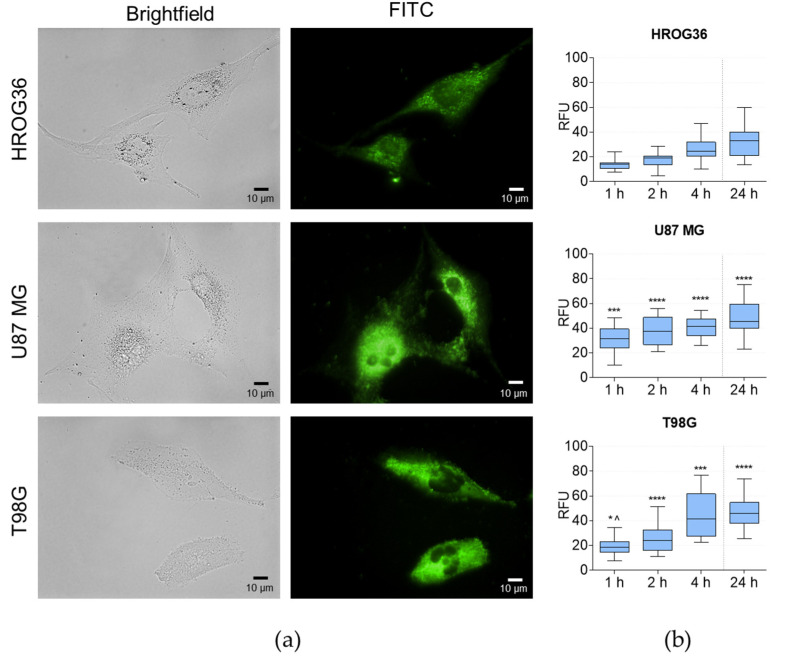
Tracking ASC-EV uptake by HROG36, U87 MG, and T98G GBM cells. GBM cells were treated with stained ASC-EVs and imaged using fluorescence microscopy. (**a**) Representative brightfield and fluorescence microscopy images of cells after 4 h treatment with fluorescently labelled ASC-EVs. Scalebar—10 μm. (**b**) Fluorescence intensity per cell corresponds to the amount of ASC-EVs internalised by GBM cells. Mann–Whitney U test; * *p* < 0.05; *** *p* < 0.001; **** *p* < 0.0001 when compared to HROG36; ^ *p* < 0.05 when compared to U87 MG.

Next, the EV uptake by GBM cells was evaluated. EVs were labelled with the SYTO RNA Select fluorescent cell dye, which selectively binds to RNA. HROG36, U87 MG, and T98G cells were incubated with the stained EVs for 24 h, taking images at 1, 2, 4, and 24 h. Green fluorescence clusters represent labelled EVs within subcellular compartments in Figure 2a. The data show that EV internalisation by GBM cells is accelerated during the first 4 h of incubation. Most ASC-EVs are internalised in the first 4 h of incubation, and prolonging treatment to 24 h barely changes fluorescence intensity (Figure 2b). However, quantitative analysis of the images revealed internalisation rate differences between distinct GBM cell lines (Figure 2b); HROG36 cells had a significantly lower ASC-EV uptake than U87 MG and T98G cells. 

**Figure 3:**
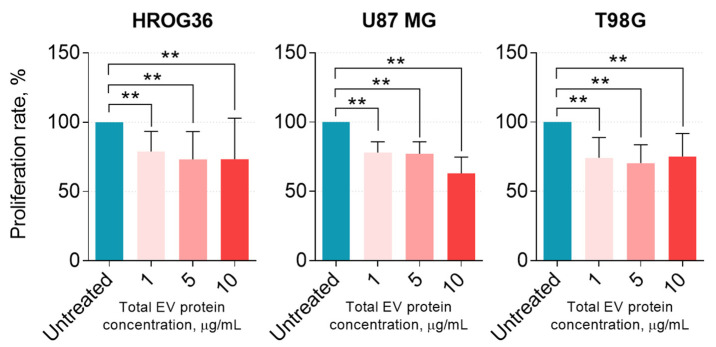
The effect of ASC-EVs on GBM cell proliferation. Changes in cell activity were determined using a resazurin-based solution, which measures the reducing power of metabolically active cells by alterations in fluorescence intensity. Mann–Whitney U test; ** *p* < 0.01.

Next, the effect of ASC-EVs on GBM cell proliferation was determined. GBM cell treatment with 1 μg/mL ASC-EVs for 24 h significantly reduced proliferation by approximately 25% (Figure 3). Further increases in ASC-EV concentration to 5 and 10 μg/mL did not further change the proliferation rate of HROG36 and T98G cells, but U87 MG proliferation with 10 μg/mL ASC-EVs dropped by approximate 35% from the control level.

### 3.3. Biomicroscopy of GBM Xenografts on CAM

**Figure 4:**
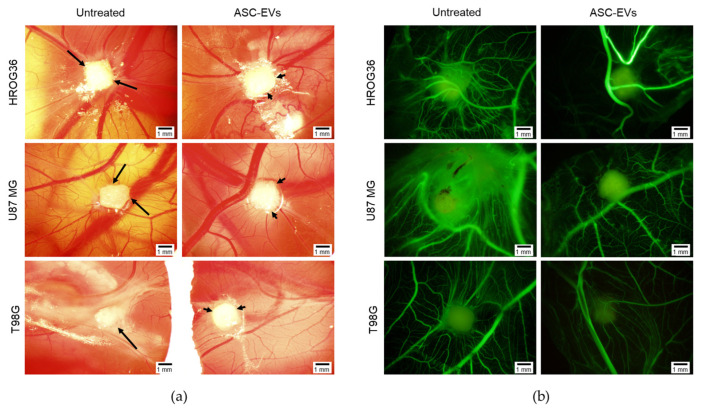
Images of GBM xenograft tumours on CAM in ovo. Following 48 h post-ASC-EVs treatment, cells were adhered to a surgical sponge and implanted onto CAMs on EDD7. Images represent xenografts on EDD12. (**a**) Untreated xenografts have an edgy outline (indicated by long arrows), whereas ASC-EV tumours have clear outlines (indicated by short arrows). (**b**) Vascular network images under the tumour after injection of fluorescent dextran images. (**a**,**b**) Scale bar—1 mm.

The CAM model was applied to investigate the ASC-EV effect on xenografted GBM. Cells were cultivated with 5 μg/mL of ASC-EVs for 48 h, trypsinised and then implanted onto CAMs. Figure 4 shows the biomicroscopy data of ASC-EV-treated and control GBM tumours on the EDD12—5 days after tumour transplantation on CAM. Biomicroscopy images revealed that untreated tumours from all cell lines had sharp, edgy outlines indicating a diffuse growth into CAM. On the contrary, ASC-EV-treated tumours appeared with clear, smooth contours representing growth on the CAM’s surface without strong adhesion or deeper penetration (Figure 4a). Untreated tumours in all study groups were surrounded by a dense vascular network (“spoked-wheel”), which was significantly diminished in ASC-EV-treated tumours (Figure 4a). The inhibitory effect of ASC-EVs on GBM tumour neovascularisation was better visible after fluorescent dextran injection into CAM’s vessel (Figure 4b). 

### 3.4. Histological Examination of CAM

**Figure 5:**
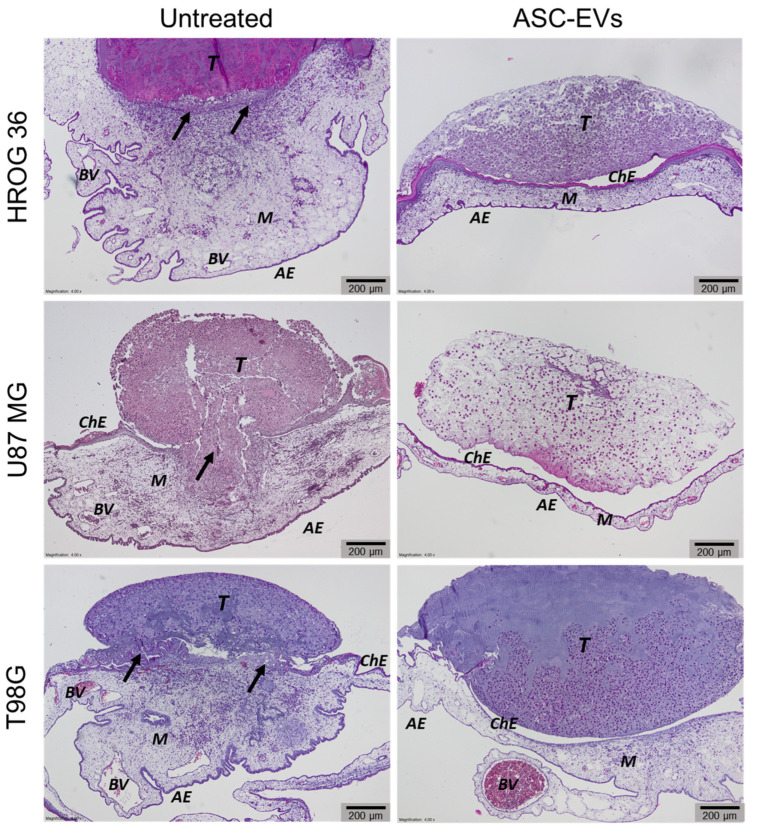
Histologic images of CAM and tumour invasion into CAM. At EDD12, CAMs were removed from the eggs, fixed, paraffin-embedded, and prepared for histological examination. Black arrowheads point towards sites of the invasion and destruction of the chorionic epithelium. ASC-EV-treated tumours did not show signs of invasion and did not penetrate through the chorionic epithelium. ChE: Chorionic epithelium, AE: Allantoic epithelium, BV: Blood vessels, M: Mesenchyme, T: Tumors. Scale bars—200 μm.

The histological examination of CAM revealed additional characteristics assessing tumour and blood vessel development (Figure 5, Table 1). In the control group, a disruption of chorionic epithelium and tumour cell invasion into CAM mesenchyme was observed. The CAM under the control tumour appeared thickened and denser due to the development of blood vessels under the tumour. Treatment with ASC-EVs significantly reduced tumour invasion into CAM incidence; ASC-EV-affected tumours in histological analysis images are on top of CAM with intact chorionic epithelium. Additionally, the CAMs under the ASC-EV-treated tumours were less thickened and had fewer blood vessels. However, a statistically significant change in blood vessel number was found only in U87 cell line samples.

### 3.5. Gene Expression Changes in GBM Cells after Treatment with ASC-Derived EVs

**Figure 6:**
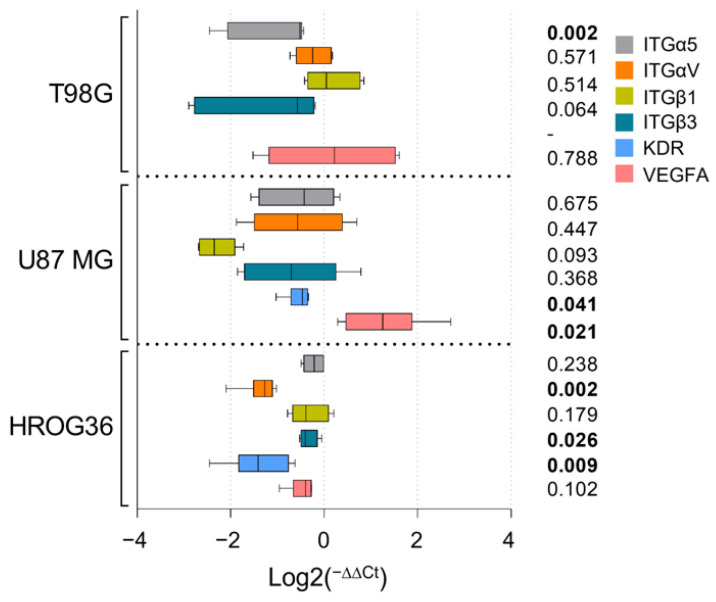
Gene expression changes in GBM cells after treatment with ASC-EVs. Data are presented as log2 Fold Change (2^−ΔΔCT^) normalised to untreated samples. Box plots with a line at the median represent summary data of 6 biological replicates. Statistical differences in gene expression values between untreated and ASC-EV treated cells were calculated by the Mann–Whitney U test; exact *p* values are given aside from the plots; bolded values highlight *p* < 0.05.

Next, we tested if the gene expression changes mediate GBM invasiveness and vascularisation suppression by ASC-EVs in CAM models. Based on several studies examining miRNA cargo in ASC-EVs (Table A2), integrin subunit alpha 5 (ITGα5), integrin subunit beta (ITGβ1), integrin subunit alpha V (ITGαV), integrin subunit beta 3 (ITGβ3), as well as vascular endothelium growth factor A (VEGFA) and its receptor gene, kinase insert domain receptor (KDR), were chosen for evaluation. The ITGα5—ITGβ1 complex is involved in tumour cell migration and invasion, and ITGβ1 plays a role in inhibiting angiogenesis. Likewise, the ITGαV—ITGβ3 complex is responsible for cell adhesion and spreading. VEGFA and KDR are central components in the VEGF signalling pathway, modulating cell migration, proliferation, survival, and vascular permeability [37,38,39]. After GBM cell treatment with 5 μg/mL ASC-EVs for 24 h, most of the selected genes were downregulated in a cell line-dependent manner (Figure 6). Such differences in intracellular processes and variability in treatment response between GBM cell lines could be expected because GBM tumours are characterised by high cellular heterogeneity [40]. ASC-EV treatment significantly downregulated ITGαV, ITGβ3, and KDR genes in HROG36 cells. In U87 MG cells, ASC-EV effects were similar to those in HROG36 except for VEGFA, which was significantly upregulated. Additionally, in ASC-EV-treated U87 MG, there was a strong trend in the suppression of ITGβ1, which plays an essential role in tumour cell adhesion. In T98G cells, ASC-EV treatment caused the significant inhibition of ITGα5. A robust trend was observed in ITGβ3 downregulation as well. We could not examine exact changes in KDR expression in T98G, as it was below the detection threshold in both untreated and ASC-EV-treated cells. To summarise, treatment with ASC-EVs tends to reduce the expression of ITGα5, ITGαV, ITGβ1, ITGβ3, and KDR genes, which could be essential for tumour cell invasion into CAM. However, it is noticeable that GBM cell cultures are different from each other, thus leading to dissimilar functional response to ASC-EVs.

## 4. Discussion

The challenges in managing GBM persist because of limited screening protocols, the aggressiveness of the tumour, and insufficient treatment choices available. However, the biotechnology sector’s rapid advancements have opened up opportunities for in-depth research and the creation of innovative medical therapies for GBM. Possible novel malignant brain tumour treatments include cell-based therapies, immunotherapies, gene therapies, and targeted therapies; nevertheless, GBM remains incurable [41]. Current possible remedies are bound to surgery, chemotherapy, radiotherapy, and, in some cases, targeted therapy, which remain insufficient to eliminate GBM tumours. Complete eradication of tumours due to cancer cell infiltration is still an unachievable task, as a 90% resection threshold without compromising functional pathways remains a desired goal. Additionally, the heterogeneity of GBM makes complete eradication impossible due to the chemo-resistance of the cells and quick recurrence and invasion in parenchyma [42]. With all the improvements in standard GBM treatment, the survival mean is approximately 15 months, with only 10% of patients living more than 5 years [43].

The emerging role of EVs in disease treatment or/and as drug carriers provide new options for possible innovative remedies. Current trends revolve around using engineered stem cell-derived EVs to deliver various anticancer agents to induce effects on cancer cells. Some studies have compared cell-based and EV-based therapies showing similar outcomes [44] and even outlined EV superiority grounding conclusions on possible immune rejections [45] and in vivo cell differentiation [46]. Thus, EV therapies could provide more benefits of cell therapy without related drawbacks. It is important to emphasize that EVs are able to cross the blood–brain barrier (BBB). Several studies have shown that EVs, even loaded with exogenous materials, are able to reach brain cells when administered intranasally or injected into the blood stream [15,44,47]. As BBB crossing remains a challenge for effective therapy of brain tumours [48], EVs are an attractive potential drug carrier which could eventually revolutionise traditional therapy approaches. However, EV application in cancer therapy raises some concerns. Evidence shows that MSC-derived exosomes affect tumour progression in both inducing and inhibiting ways. Herein, we report that ASC-EVs could provide tumour-suppressive effects in vitro and in ovo. 

ASC-EV isolates’ characterisation according to the origin-indicating markers [33] revealed high amounts of non-tissue-specific transmembrane tetraspanins CD6 and CD81, essential in exosome biogenesis [49,50]. A scaffold protein syntenin-1, responsible for directing endocytosed syndecans and syndecan cargo to budding endosomal membranes [51], was also present in the samples confirming the presence of exosomes. The presence of integrin VLA-1, characteristic of the plasma membrane, indicates that part of the ASC-EVs were ectosomes. Low traces of Cyt c were also present in ASC-EV samples, indicating that some particles originated from non-endosomal and non-plasma membrane intracellular compartments. ASC-EVs were swiftly internalised by GBM cells and caused proliferation-suppressing effects. However, the internalisation and proliferation modulation capacity and gene expression response profile varied between the GBM cell lines. This inconsistency between different GBM cell lines could be explained by cancer cell plasticity and heterogeneity of GBM tumours [40]. Experiments with CAM provided functional evidence for the antitumour effects of ASC-EVs. Tumours formed after treatment with ASC-EVs showed a significantly lower invasion rate when compared to untreated ones. Noteworthy, ASC-EV-affected U87 MG tumours also had significantly weaker neo-angiogenesis. Interestingly, gene expression data revealed that ASC-EV treatment inhibits KDR but promotes VEGFA expression in the cells of this line. The fact that U87 MG tumours had less pronounced vascular networks in the presence of ASC-EVs provides additional evidence that KDR plays a vital role in tumour vascularization. This finding supports the application of KDR inhibition as a strategy to develop selective and specific anticancer agents [52]. In addition to blood vessel modulating effects, ASC-EVs caused significant downregulation of ITGα5 in T98G cells. ITGa5 forms a complex with ITGβ1 and is responsible for tumour cell migration and invasion. According to a recent study, this gene is involved in essential oncological pathways and is responsible for typical genomic alterations in gliomas, including the tumour immune microenvironment formation [53]. ITGβ1 showed a strong trend to be downregulated by ASC-EVs in U87 MG. It is important to mention that ITGαV and ITGβ3 complexes play a significant role in cell adhesion and spreading [54], and both of these genes were found downregulated in HROG36 cells. ASC-EVs carry a broad spectrum of functional miRNAs related to tumorigenicity and tumour suppression [55,56,57]. In addition, most miRNAs found in ASC-EVs can target ITGα5 and ITGαV [58]. A study published by Morandi and colleagues revealed that ITGα5 and ITGαV diversely regulate the proliferation and adipogenic differentiation of human adipose-derived stem cells. This could explain the abundance of ITGαV-associated miRNAs in ASC-EV cargo [59].

In our study, ASC-EVs were well internalised by GBM cells and reduced cell migration and proliferation. However, it is crucial to emphasise inconsistencies between studies, even concerning EVs from MSCs of the same origin. Qin and co-authors have demonstrated that bone marrow-derived MSC-EVs promote osteosarcoma tumorigenesis [60]. Their study focused on miR-208a, which induced cell migration and invasiveness by modulating the programmed cell death 4 (PDCD4) gene and extracellular signal-regulated kinase 1/2 signalling pathway. From a different perspective, bone marrow-derived MSC-EVs cause significant downregulation of VEGF in breast cancer cells via miR-100 [61]. MSCs recruited to tumour sites by various microenvironmental factors, e.g., nutrient deprivation, might change their phenotype, causing alterations in paracrine mediators, including the EVs. For example, miR-1587 harboured by glioma-associated MSC-EVs leads to the proliferation of glioma cells [28]. Another group has discovered that exosomes produced by bone marrow-derived MSCs from multiple myeloma environment promote tumour growth, but those from normal bone marrow-derived MSCs cause a completely opposite effect [62]. The researchers revealed that EVs from multiple myeloma-affected MSCs have less tumour-suppressing miR-15a and increased levels of oncogenic proteins, cytokines, and adhesion molecules. Our results demonstrated that ASC-EVs do not exhibit tumorigenic properties in GBM cultures, which is essential for application as drug-delivery vehicles. In addition, the evidence that ASC-EVs can reduce GBM proliferation, invasiveness, and neoangiogenesis suggests them as adjuvant therapy for treating this disease.

## 5. Conclusions

ASC-EVs significantly reduce the proliferation of cultured GBM cells and suppress tumour invasiveness and vascularisation in ovo, indicating they are safe to use as anticancer drug carriers and might be exploited as therapeutics. However, the exact mechanism of ASC-EVs’ anticancer activity remains to be determined.

## Figures and Tables

**Table 1:** Percentage of tumours invaded into CAM, thickness calculations of CAM, and the number of blood vessels in CAM under the tumour.

Study Group	n	Invasion %	CAM Thickness Median (Range)	Number of Blood Vessels Median (Range)
HROG36—Untreated	10	90 ^a^	199.9 (109.5–585.6)	22 (6–51)
HROG36—ASC-EVs	12	33 ^a^	278.6 (114.2–511.0)	14.5 (7–45)
U87 MG—Untreated	9	80 ^b^	354.0 (190.5–516.6) ^d^	24 (16–38) ^e^
U87 MG—ASC-EVs	11	9 ^b^	134.7 (66.7–345.4) ^d^	18 (3–25) ^e^
T98G—Untreated	12	83 ^c^	248.1 (60.11–968.2)	28 (3–45)
T98G—ASC-EVs	6	17 ^c^	229.1 (193.5–527.3)	19.5 (10–36)

Groups indicated by the same superscript letter were compared. ^a^ *p* = 0.0071, ^b^ *p* = 0.0002, ^c^ *p* = 0.067 (Chi square test) ^d^ *p* = 0.0015, ^e^ *p* = 0.0015 (Mann–Whitney U test)

## Data Availability

The raw data supporting the conclusions of this manuscript will be made available by the authors, without undue reservation, to any qualified researcher.

## Appendix A

**Table A1:** Primer sequences used in RT-qPCR.

Target	Sequence
β-Actin	F: AGAGCTACGAGCTGCCTGAC R: AGCACTGTGTTGGCGTACAG
GAPDH	F: TCAAGATCATCAGCAATGCCT R: CATGAGTCCCACGATACC
ITGα5	F: GTGGCCTGCATCAACCTTAGC R: TTCTGGATGAGCAGGGTCTGG
ITGβ1	F: GCGTGCAGGTGCAATGAAGG R: ACAAACACACTGTCCGCAGACG
ITGαV	F: GCGTATCTGCGGGATGAATCTG R: AATGTTAGCAGGCGTGAACTGG
ITGβ3	F: TTGGAGACACGGTGAGCTTCAG R: CTGGCAGGCACAGTCACAATC
VEGFA	F: AGGAGTCCAACATCACCATGCA R: CAAGGCCCACAGGGATTTTCTTG
KDR	F: CATCGCGAAAGTGTATCCACAGG R: TTCAAAGGGAGGCGAGCATC

**Table A2:** List of miRNAs enriched in primary ASC-EVs with targeting capabilities for ITGα5, ITGαV, KDR, and VEGFA. miRNA data reported by Alonso-Alonso et al.

Gene Name	Gene Symbol	MiRNAs Carried by ASC-EVs	TarBase v8 Prediction Score	MiRNAs Carried by ASC-EVs	TarBase v8 Prediction Score
Integrin alpha subunit 5	ITGα5	hsa-miR-98-5p	0.616	hsa-miR-92b-3p	0.999
		hsa-miR-130a-3p	0.777	hsa-miR-29b-3p	0.508
		hsa-miR-148a-3p	0.998	hsa-miR-29c-3p	0.53
		hsa-miR-148b-3p	0.998	hsa-miR-32-5p	0.996
		hsa-miR-152-3p	0.998	hsa-miR-326	0.968
		hsa-miR-181a-2-3p	0.462	hsa-miR-330-5p	0.999
		hsa-miR-22-3p ^b^	0.704	hsa-miR-423-5p	0.853
		hsa-miR-22-5p	0.828	hsa-miR-425-5p	0.667
		hsa-miR-25-3p	0.992	hsa-miR-766-3p	0.473
		hsa-miR-92a-3p	0.999		
Integrin alpha subunit V	ITGαV	hsa-let-7a-3p	0.999	hsa-miR-30d-5p	0.519
		hsa-let-7a-5p	0.999	hsa-miR-30e-5p	0.52
		hsa-let-7b-5p	0.472	hsa-miR-32-5p	0.995
		hsa-let-7c-5p	0.472	hsa-miR-320b	0.464
		hsa-let-7d-5p	0.547	hsa-miR-320c	0.464
		hsa-let-7e-5p	0.512	hsa-miR-34a-3p	0.621
		hsa-let-7f-5p	0.477	hsa-miR-361-5p	0.913
		hsa-let-7g-5p	0.477	hsa-miR-374a-5p	0.823
		hsa-miR-98-5p	0.477	hsa-miR-493-3p	0.586
		hsa-miR-132-3p	0.682	hsa-miR-501-3p	0.459
		hsa-miR-142-5p	0.997	hsa-miR-501-5p	0.459
		hsa-miR-192-3p	0.829	hsa-miR-502-3p	0.455
		hsa-miR-192-5p	0.613	hsa-miR-548d-5p	0.863
		hsa-miR-23a-3p ^b^	0.503	hsa-miR-582-3p	0.717
		hsa-miR-25-3p	0.989	hsa-miR-200b-3p	0.805
		hsa-miR-92a-3p	0.999	hsa-miR-200c-3p	0.798
		hsa-miR-30a-5p	0.517	hsa-miR-429 ^a^	0.798
		hsa-miR-30b-5p	0.529	hsa-miR-545-5p ^a^	0.867
		hsa-miR-30c-5p	0.528		
Kinase Insert Domain Receptor	KDR	hsa-miR-16-5p	0.873	hsa-miR-23a-3p ^b^	0.61
		hsa-miR-21-3p	0.732	hsa-miR-200c-3p	0.999
Vascular Endothelial Growth Factor A	VEGFA	hsa-miR-15a-5p	0.848	hsa-miR-9-5p ^a^	0.55
		hsa-miR-205-5p	0.752	hsa-miR-23b-3p ^c^	0.613

^a^ Additionally observed by Eirin et al. ^b^ Additionally observed by Mitchel et al. ^c^ Observed by Mithcel et al.
